# Vibrotactile Stimulation Based on the Fundamental Frequency Can Improve Melodic Contour Identification of Normal-Hearing Listeners With a 4-Channel Cochlear Implant Simulation

**DOI:** 10.3389/fnins.2019.01145

**Published:** 2019-10-29

**Authors:** Xin Luo, Lauren Hayes

**Affiliations:** ^1^College of Health Solutions, Arizona State University, Tempe, AZ, United States; ^2^School of Arts, Media and Engineering, Arizona State University, Tempe, AZ, United States

**Keywords:** cochlear implant, music perception, melodic contour, tactile aid, vibration perception, multisensory integration

## Abstract

Cochlear implant (CI) users’ poor speech recognition in noise and music perception may be both due to their limited access to pitch cues such as the fundamental frequency (F0). Recent studies showed that similar to residual low-frequency acoustic hearing, vibrotactile presentation of the F0 significantly improved speech recognition in noise of CI users. The present study tested whether F0-based vibrotactile stimulation can improve melodic contour identification (MCI) of normal-hearing listeners with acoustically simulated CI processing. Each melodic contour consisted of five musical notes with one of nine contour patterns (rising, falling, or flat in each half of the contour). The F0 of the middle note was 220 or 880 Hz, and the frequency intervals between adjacent notes were 1, 3, or 5 semitones. The F0 of each note was extracted in real time and transposed to a vibration frequency centered around 110 Hz at the right forearm top. MCI was tested in five experimental conditions (with a 4- or 8-channel CI simulation alone, vibrotactile stimulation alone, and 4- or 8-channel CI simulation plus vibrotactile stimulation), each after the same amount of brief training was provided. Results showed that discrimination of vibrotactile stimuli significantly improved from chance to near perfect as the vibration frequency interval increased from 0.25 to 3 semitones. The MCI performance with vibrotactile stimulation alone was similar to that with the 4-channel CI simulation alone, but was significantly worse than that with the 8-channel CI simulation alone. Significant improvement in MCI performance with the addition of vibrotactile stimulation was only found with the 4-channel CI simulation when the middle F0 was 880 Hz and when the frequency intervals were 3 or 5 semitones. The improvement in MCI performance with than without vibrotactile stimulation was significantly correlated with the baseline MCI performance with 4-channel CI simulation alone or with the MCI performance difference between vibrotactile stimulation and 8-channel CI simulation. Therefore, when the simulated or real CI performance is relatively poor, vibrotactile stimulation based on the F0 may improve MCI with acoustic CI simulations and perhaps in real CI users as well.

## Introduction

Cochlear implant (CI) is widely used to restore hearing sensation to profoundly deaf people through electrical stimulation of surviving auditory neurons. It is remarkable that the majority of CI users can have good speech recognition in quiet with only slowly-varying temporal envelope cues from a small number of frequency channels (Wilson and Dorman, 2008). However, the crude mimicking of normal peripheral auditory processing by current CI systems makes music perception extremely challenging (e.g., Limb and Roy, 2014). Although the coarse temporal features of music (e.g., rhythm, tempo, and meter) are well preserved for CI users, pitch perception that requires spectro-temporal fine structure cues is much worse in CI users than in normal-hearing (NH) listeners. Place-of-stimulation cues for pitch perception with CIs are limited by the use of only 12–22 implanted electrodes, the current spread of electrical stimulation, and the frequency-to-place mismatch due to shallow insertion of the electrode array. The spectral resolution needed for place-pitch perception is also affected by the neural survival and electrode-to-neuron distance (Bierer, 2010). On the other hand, CI users’ pitch perception based on the pulse rate of an electrical pulse train or the amplitude modulation frequency of a constant-rate electrical pulse train has been shown to saturate around 300 Hz (i.e., rates higher than 300 Hz do not lead to higher pitch perception). It seems that 300 Hz is the auditory nerve entrainment limit with electrical stimulation, above which the all-order inter-spike interval distributions more closely reflect the maximum sustained neural response rate rather than the F0 subharmonics of acoustic input. As such, CI users have poorer-than-normal perception of both the directions (i.e., contours) and sizes (i.e., intervals) of pitch changes in musical melodies (e.g., Galvin et al., 2007; Luo et al., 2014b).

For CI users with residual low-frequency acoustic hearing in the non-implanted ear, pitch cues may be better accessed through the use of a hearing aid in conjunction with the CI (i.e., binaural bimodal hearing). The residual acoustic hearing is typically available for frequencies up to 1000 Hz and thus adds complementary low-frequency pitch cues such as the F0 and lower harmonics to CI-mediated electric hearing. Compared to CI alone, bimodal hearing has been shown to significantly improve familiar melody recognition (Kong et al., 2005; Gfeller et al., 2006) and melodic contour identification (Crew et al., 2015) by 20–30%, and the speech reception threshold in noise by 1–5 dB. Although promising, the bimodal benefits to speech and music perception are only available for those with residual acoustic hearing. However, the proportion of CI candidates with residual acoustic hearing is still low (e.g., only 9% in Verschuur et al., 2016) even after significant increases over the years.

Recently, vibrotactile stimulation has been proposed as an alternative way to deliver low-frequency acoustic cues to CI users without residual acoustic hearing (Huang et al., 2017; Fletcher et al., 2018). This idea was based on the fact that the most sensitive frequency range of touch sense is below 500 Hz (Verrillo, 1985), similar to the low-frequency range of residual acoustic hearing that accounts for the majority of bimodal benefits (Zhang et al., 2010). Unlike traditional tactile aids that were designed as an alternative to CIs for auditory rehabilitation for profound deafness, electro-tactile stimulation was aimed to combine CI-mediated electric hearing with low-frequency vibrotactile stimulation. In this application, important low-frequency acoustic cues identified by studies of bimodal hearing (e.g., Brown and Bacon, 2009) were presented via vibrotactile stimulation to help improve CI performance. For example, Huang et al. (2017) found that vibrotactile stimulation based on the F0 of clean speech significantly improved the speech reception threshold in steady-state, speech-shaped noise of real CI users by 2 dB on average. Also, Fletcher et al. (2018) used the temporal envelope and voicing information of noisy speech for vibrotactile stimulation and found significantly better speech recognition in multi-talker, speech-babble noise with than without vibrotactile stimulation of NH listeners listening to an acoustic CI simulation. The improvement in speech recognition with the addition of vibrotactile stimulation at the speech reception threshold was on average 10%.

The first aim of the present study was to test whether F0-based vibrotactile stimulation can improve melodic contour identification (MCI; Galvin et al., 2007) of NH listeners listening to acoustic CI simulations. As an important aspect of music perception, MCI was tested with CI simulations alone, vibrotactile stimulation alone, and CI simulations plus vibrotactile stimulation. The MCI test was ideal for repeated testing in different conditions because the effect of memory recall was reduced by testing a large number of novel melodic contours. It also allowed for a systematic manipulation of the pitch ranges and intervals of melodic contours to clarify the mechanisms of MCI. CI simulations were used to test NH listeners, so that the spectral resolution of acoustic stimulation can be precisely controlled without differences between individual CI systems and patient-related confounds such as the neural survival and electrode placement. A 4- or 8-channel noise-band vocoder was used to simulate the number of effective frequency channels in real CI users (Friesen et al., 2001). To represent real-world applications, the F0 of each musical note was extracted in real time with low computational cost and was used by a compact, wearable device to produce vibrotactile stimulation at the top of the right forearm of participants. Different pitch ranges of melodic contours were transposed to the low frequency range of vibration. It was hypothesized that vibrotactile stimulation would improve the MCI performance of NH listeners listening to acoustic CI simulations, but the amount of improvement may vary with the pitch ranges and intervals of melodic contours, as well as the number of frequency channels in the CI simulation.

Large variability across participants in terms of the MCI improvement with simulated electro-tactile stimulation compared to CI simulations alone was also expected, similar to that observed for speech recognition in noise (Huang et al., 2017; Fletcher et al., 2018). The second aim of the present study was to understand the factors contributing to the variability in MCI improvement with simulated electro-tactile stimulation. Factors of interest were the perceptual sensitivity to vibrotactile stimulation, MCI performance with vibrotactile stimulation alone, MCI performance with CI simulations alone, and MCI performance difference between vibrotactile stimulation and CI simulations (i.e., the MCI performance with vibrotactile stimulation alone minus that with the CI simulations alone). Correlations between these outcome measures and the MCI improvement with simulated electro-tactile stimulation were analyzed. The hypothesis was that similar or more salient melodic contour cues from vibrotactile stimulation than from CI simulations are needed for MCI improvement with simulated electro-tactile stimulation.

## MATERIALS AND METHODS

### Participants

Eight young adult NH listeners (all female, age range: 19–32 years, mean age: 22 years) participated in this study. All participants had hearing thresholds lower than 20 dB HL at octave frequencies from 125 to 8000 Hz in both ears. None of them had extensive musical training. All participants gave informed consent and were compensated for their participation. This study was approved by the Institutional Review Board of Arizona State University.

### Psychophysics of Vibrotactile Stimulation

#### Vibrotactile Device

**FIGURE 1:**
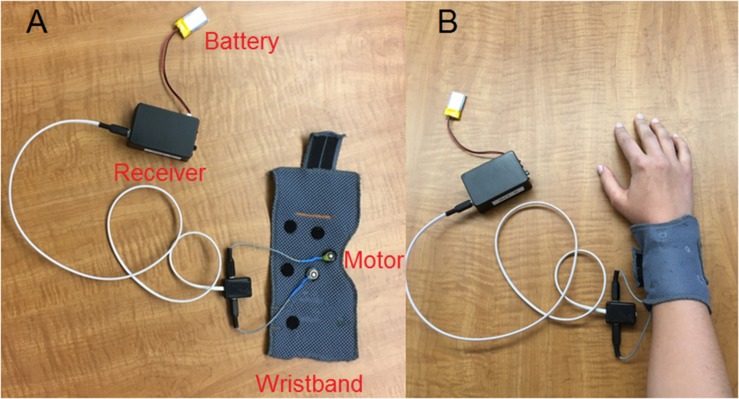
Vibrotactile device used in the present study with the different parts labeled **(A)** and when the wristband was put around the right arm of a participant **(B)**.

A wearable vibrotactile device (Figure 1A) was used in the present study. A Precision Microdrives^TM^ pancake-shape vibration motor with a 10 mm diameter and a 3 mm length (similar to those used in cell phones) was attached to a wristband using Velcro. A battery-powered wireless receiver (i.e., the Sense/Stage MiniBee system^1^) was controlled by customized Max/MSP^2^ software to vibrate the motor tangentially to the skin. For the Eccentric Rotating Mass (ERM) vibration motor used in the present study, the vibration waveform is a sinusoidal function of time. According to the datasheet^3^, the motor has an operating range of input voltage from 0.7 to 3.7 v. The vibration frequency and amplitude both increase (up to 220 Hz and 1.62 g, respectively) with increasing input voltage, thus prohibiting the possibility for decoupling the vibration frequency and amplitude. During testing, participants put the wristband around their right arm firmly and comfortably, and rested their right arm on a desk in a sound booth with the hand palm-side down (Figure 1B). Previous studies of electro-tactile stimulation (Huang et al., 2017; Fletcher et al., 2018) sent vibrotactile stimulation to the index fingertip, while the present study used the top of the right forearm, which was a more suitable site of vibrotactile stimulation for real-world applications due to its less interference with hand movements. Our pilot study showed that the forearm top had slightly but not significantly higher vibration detection thresholds than the index fingertip.

#### Vibration Detection

Each participant’s absolute and differential sensitivity to vibrotactile stimulation was measured using classical psychophysical tests. The method of limits was used to estimate the vibration detection threshold (i.e., the lowest input voltage that generated a perceivable vibration). In an ascending sequence, a 500 ms vibration started with a subthreshold level and the input voltage increased in steps of 0.013 v until the vibration was detected. The last undetected and the first detected voltages were averaged as the endpoint of the ascending sequence. In a descending sequence, the vibration started with a supra-threshold level and the input voltage decreased in steps of 0.013 v until the vibration was no longer detected. The last detected and the first undetected voltages were averaged as the endpoint of the descending sequence. Three ascending and three descending sequences were tested alternately in counter-balanced order across participants. The endpoints of these ascending and descending sequences were averaged as the estimated vibration detection threshold.

#### Vibration Discrimination

The psychometric function of vibration discrimination was measured using a two-alternative, forced-choice (2AFC) task. There were two 500 ms vibrations separated by a 100 ms gap in each trial. The two vibration frequencies were centered on 110 Hz (which was the middle of the vibration frequency range) and differed by 0.25, 0.5, 1, 2, and 3 semitones. The two corresponding vibration amplitudes were centered on 0.5 g and differed by 0.01, 0.02, 0.04, 0.08, and 0.12 g, respectively. The order of presentation was random for the two vibrations and participants were asked to select the vibration with a higher frequency, although the vibration amplitudes may also be used to perform the discrimination task. Feedback regarding the response correctness was not provided. The five vibration frequency intervals were each tested 10 times in random order, resulting in a total of 50 trials in a session. Three sessions were completed. The average percent correct score of vibration discrimination was calculated for each frequency interval.

### Melodic Contour Identification

#### Melodic Contours

**FIGURE 2:**
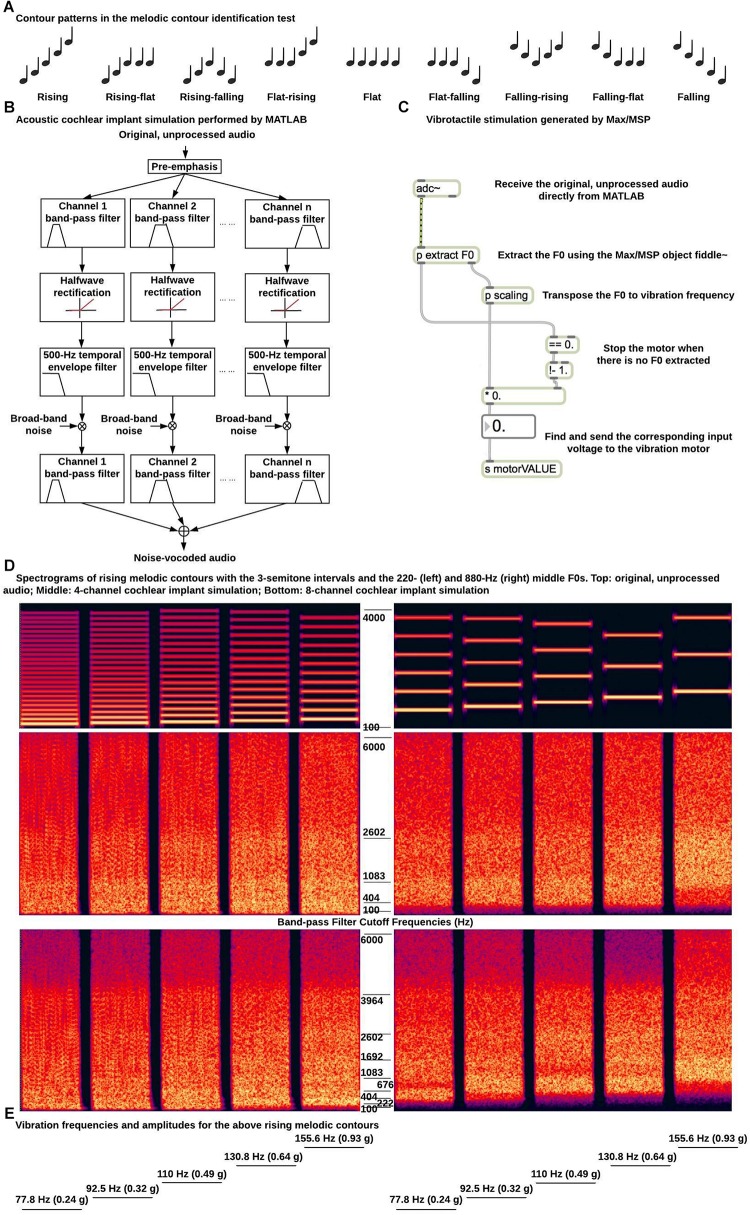
Example stimuli and signal processing for the melodic contour identification test. **(A)** Nine contour patterns used in the melodic contour identification test; **(B)** Diagram of acoustic simulation of CI processing; **(C)** Diagram of signal processing for vibrotactile stimulation; **(D)** Spectrograms of rising melodic contours with the 3-semitone intervals and the 220 (left) and 880 Hz (right) middle F0s (top, original, unprocessed audio; middle, 4-channel CI simulation; bottom, 8-channel CI simulation). The band-pass filter cutoff frequencies for the CI simulations are listed next to the spectrograms. **(E)** Vibration frequencies and amplitudes for the rising melodic contours in **(D)**.

The MCI test (Galvin et al., 2007) was used in the present study. Each melodic contour had five 500 ms musical notes with 100 ms gaps in between. Each note was a harmonic complex tone, which had all harmonics in sine phase up to 4000 Hz and a spectral slope of −8 dB/octave. Each note also had 20 ms raised-cosine onset and offset ramps. As shown in Figure 2A, the F0-change direction (i.e., rising, flat, or falling) within the first half of the melodic contour (i.e., from the first to the third note) was independent from that within the second half (i.e., from the third to the fifth note), resulting in a total of nine contour patterns (i.e., rising, rising-flat, rising-falling, flat-rising, flat, flat-falling, falling-rising, falling-flat, and falling). The F0 of the middle (or the third) note was 220 (A_3_) or 880 Hz (A_5_) to test MCI in different pitch ranges. The F0 differences between adjacent notes were 1, 3, or 5 semitones to test MCI with different pitch intervals. All 54 melodic contours (2 middle F0s × 3 interval sizes × 9 contour patterns) were generated with a 22,050 Hz sampling rate and a 16-bit resolution in MATLAB.

#### Acoustic CI Simulations

For acoustic stimulation, the melodic contours were processed in MATLAB by a 4- or 8-channel noise-band vocoder to simulate CI processing (Shannon et al., 1995; Figure 2B). The musical notes were first pre-emphasized by a first-order Butterworth high-pass filter at 1200 Hz. This pre-emphasis is used in CI systems to flatten the long-term average spectrum and enhance the perception of low-intensity, high-frequency spectral components. The musical notes were then filtered into 4 or 8 channels by fourth-order Butterworth band-pass filters. The overall frequency range of analysis was from 100 to 6000 Hz and the band-pass filter cut-off frequencies (listed in Figure 2D) were calculated using the Greenwood (1990) function so that the filters were evenly spaced in terms of their cochlear positions. Each band-pass filtered signal was half-wave rectified and low-pass filtered by a fourth-order Butterworth filter at 500 Hz to extract the temporal envelope. The 500 Hz temporal envelope was able to preserve the temporal periodicity cues for pitch perception of melodic contours with the 220 Hz but not with the 880 Hz middle F0. A broad-band noise was amplitude modulated by the extracted temporal envelope and filtered by the band-pass filter of the channel. The amplitude-modulated noise bands of all channels were added together to generate the noise-vocoded melodic contours. Figure 2D shows the spectrograms of the rising melodic contours with the 3-semitone intervals and the 220 and 800 Hz middle F0s before and after the 4- or 8-channel CI simulation. A JBL loudspeaker placed 1 m in front of the participant in the sound booth was used to present the noise-vocoded melodic contours at a root-mean-square level of 65 dB SPL in conditions with acoustic CI simulations.

#### Vibrotactile Stimulation

The vibrotactile device described in the previous section was used to generate F0-based vibrations at the top of the right forearm in conditions with vibrotactile stimulation. As shown in Figure 2C, a Max/MSP object named fiddle∼ (Puckette et al., 1998) received the original, unprocessed melodic contours from MATLAB through direct internal audio routing and extracted the F0 of each note based on spectral analysis in real time. Each frame of 1024 samples was zero-padded to perform a 2048-point Fast Fourier Transform. Up to 20 spectral peaks were used to estimate the F0 with maximum likelihood (i.e., the harmonics of the estimated F0 should best match the spectral peaks). At least four spectral peaks should be present or the total power of the contributing peaks should be at least 0.01 of the signal power. Otherwise, no pitch was detected. A new F0 estimation was made every 512 samples. The F0 estimation was found to be accurate for all the musical notes used in the melodic contours. The estimated F0s were scaled to the range of vibration frequencies (i.e., up to 220 Hz). As mentioned, 220 Hz was the typical upper frequency limit of the ERM motor used in this study and frequencies below 220 Hz were within the most sensitive frequency range of touch sense (Verrillo, 1985). Specifically, F0s from 110 to 440 Hz (i.e., those of the melodic contours with the 220 Hz middle F0) were divided by two, while F0s from 440 to 1760 Hz (i.e., those of the melodic contours with the 880 Hz middle F0) were divided by eight to determine the vibration frequencies. The corresponding input voltages to the vibration motor were then found using the datasheet of the motor. Figure 2E shows the vibration frequencies (and the covaried vibration amplitudes) for the rising melodic contours with the 3-semitone intervals and the 220 and 880 Hz middle F0s. Note that after frequency transposition, melodic contours with the two different middle F0s had the same vibration frequencies and amplitudes.

#### Testing Procedure

MCI was first tested with only acoustic presentation of the original, unprocessed musical notes. The purpose of this baseline test was to familiarize participants with the testing procedure and make sure that each participant had near perfect performance with the original acoustic stimuli. In each trial, one of the 54 melodic contours was randomly selected for presentation without replacement and participants were asked to identify the contour pattern by clicking on one of the nine response buttons with the corresponding contour pictures. Feedback was not provided regarding the response correctness. The percent correct score of MCI was recorded for one run of the baseline test.

MCI was then tested in five experimental conditions (i.e., with the 4- or 8-channel CI simulation alone, vibrotactile stimulation alone, and 4- or 8-channel CI simulation plus vibrotactile stimulation) in random order. Before each experimental condition, a brief training was conducted to familiarize participants with the corresponding stimulation condition. Different from the testing stimuli, the training stimuli were melodic contours with a 440 Hz middle F0 and the pitch intervals between adjacent notes were 2 or 4 semitones (i.e., a total of 18 melodic contours). Accordingly, the F0s of the training stimuli were divided by a different factor (four) to determine the vibration frequencies. The training procedure was the same as the testing procedure, except that visual feedback regarding the response correctness was provided after each trial and the melodic contour was presented again with the correct response highlighted after each incorrect trial. Two runs of training with feedback were completed before two runs of testing without feedback in each experimental condition. The average MCI score was calculated for the two runs of testing.

#### Statistical Analyses

As mentioned, our main hypothesis was that the benefits of vibrotactile stimulation to overall MCI scores, those for different middle F0s, and those for different interval sizes may rely on the number of channels in CI simulations. To explore the potential interaction between vibrotactile stimulation and channel number, separate repeated-measures analyses of variance (RM ANOVAs) were used to analyze the overall MCI scores, those for different middle F0s, and those for different interval sizes with the 4- or 8-channel CI simulation with or without vibrotactile stimulation (i.e., in four of the five stimulation conditions). Another hypothesis was that the relative salience of MCI cues from vibrotactile stimulation and CI simulations alone may determine the efficacy of multi-sensory integration. Therefore, the overall MCI scores, those for different middle F0s, and those for different interval sizes were compared across the conditions with vibrotactile stimulation alone and 4- or 8-channel CI simulation alone (i.e., in three of the five stimulation conditions) using separate RM ANOVAs. *Post hoc* Bonferroni *t*-tests were used for pair-wise comparisons following the various RM ANOVAs.

## Results

### Vibration Detection and Discrimination

The vibration detection thresholds of different participants ranged from 0.71 to 0.82 v, which were slightly higher than the lower end of the motor operating range (0.7 v). It was also found that the higher end of the motor operating range (3.7 v) generated a strong but comfortable vibration for all participants. As such, the whole motor operating range can be used to encode low-frequency acoustic cues via vibrotactile stimulation.

**FIGURE 3:**
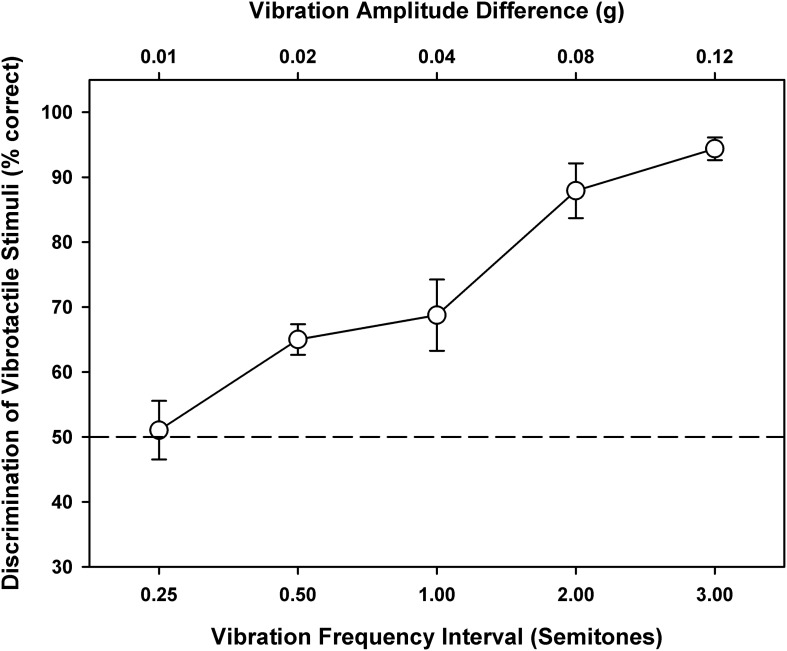
Vibration discrimination performance as a function of the vibration frequency interval (on the bottom axis) or vibration amplitude difference (on the top axis). Symbols represent the mean, while error bars represent the standard error across participants. The horizontal dashed line indicates the chance performance of 50% correct.

Figure 3 shows the percent correct scores of vibration discrimination as a function of the interval size between vibration frequencies. The vibration amplitude differences corresponding to the vibration frequency intervals are also shown on the top axis, because both vibration frequencies and amplitudes may be used to perform the discrimination task. As the frequency interval increased from 0.25 to 3 semitones, the vibration discrimination performance improved from chance to near perfect. A one-way RM ANOVA showed that the vibration discrimination performance was significantly affected by the frequency interval size [*F*_(4, 28)_ = 24.29, *p* < 0.001]. *Post hoc* Bonferroni *t*-tests showed that all pairwise comparisons of vibration discrimination performance between the interval sizes were significant (*p* < 0.02), except for 0.25 vs. 0.5, 0.5 vs. 1, and 2 vs. 3 semitones (*p* > 0.10). From the psychometric function of vibration discrimination, the vibration discrimination threshold with a 71% correct score (similar to that measured with a two-down/one-up adaptive procedure) was estimated to be 1.1 semitones (i.e., a just noticeable difference of 7 Hz around 110 Hz). As such, the frequency changes between adjacent notes in the melodic contours (i.e., 1, 3, or 5 semitones) should be reliably perceived via vibrotactile stimulation.

### Melodic Contour Identification

#### Overall MCI Scores

**FIGURE 4:**
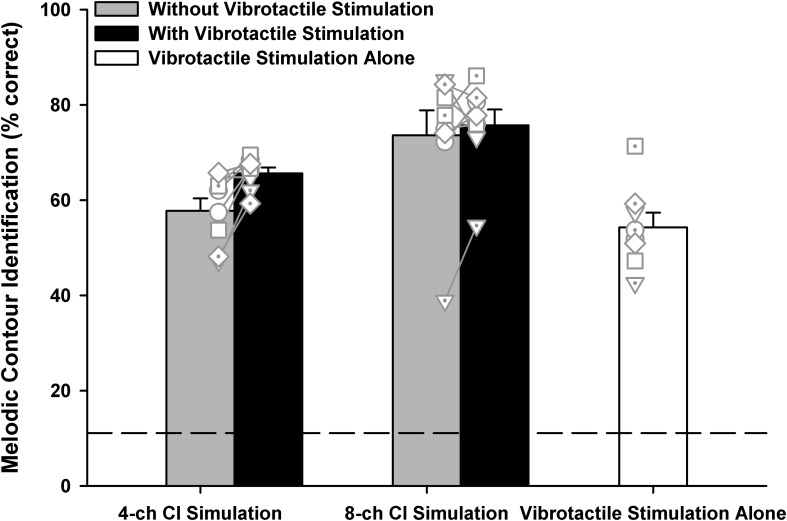
Overall melodic contour identification scores with the 4- or 8-channel CI simulation (left two and middle two bars, respectively) with or without vibrotactile stimulation (black and gray bars, respectively) or with vibrotactile stimulation alone (white bar). Vertical bars represent the mean, while error bars represent the standard error across participants. Individual data points are shown by different symbols with or without dots. The horizontal dashed line indicates the chance performance of 11% correct.

One participant scored 87% while the others scored 100% in the baseline MCI test with acoustically presented original notes. Figure 4 shows the overall MCI scores in the five experimental conditions. A one-way RM ANOVA was used to compare the performance with vibrotactile stimulation alone (white bar) to those with the 4- or 8-channel CI simulation alone (gray bars). There was a significant effect of experimental condition on the MCI performance [*F*_(2, 14)_ = 12.58, *p* < 0.001]. *Post hoc* Bonferroni *t*-tests showed that vibrotactile stimulation alone produced similar MCI performance as the 4-channel CI simulation alone (*p* = 1.00). However, the MCI performance was significantly worse with vibrotactile stimulation alone than with the 8-channel CI simulation alone (*p* = 0.001).

To test whether the MCI performance with 4- or 8-channel CI simulation was better with than without simultaneous vibrotactile stimulation (black and gray bars, respectively), a two-way RM ANOVA with channel number (4 or 8) and vibrotactile stimulation (on or off) as the two factors was conducted on the MCI performance. Note that the vibrotactile stimulation was perceived to be synchronized with the acoustic stimulation by all participants. It was found that both the channel number [*F*_(1, 7)_ = 12.48, *p* = 0.01] and vibrotactile stimulation [*F*_(1, 7)_ = 5.64, *p* = 0.04] significantly affected the MCI performance. The two factors did not significantly interact with each other [*F*_(1, 7)_ = 3.80, *p* = 0.09]. *Post hoc* Bonferroni *t*-tests showed that the MCI performance was significantly better with 8 than with 4 channels (*p* < 0.03), whether or not vibrotactile stimulation was added to the CI simulations. On the other hand, simultaneous vibrotactile stimulation significantly improved the MCI performance with 4-channel (*p* = 0.009) but not with 8-channel CI simulation (*p* = 0.43).

#### Detailed MCI Scores for Different Middle F0s

**FIGURE 5:**
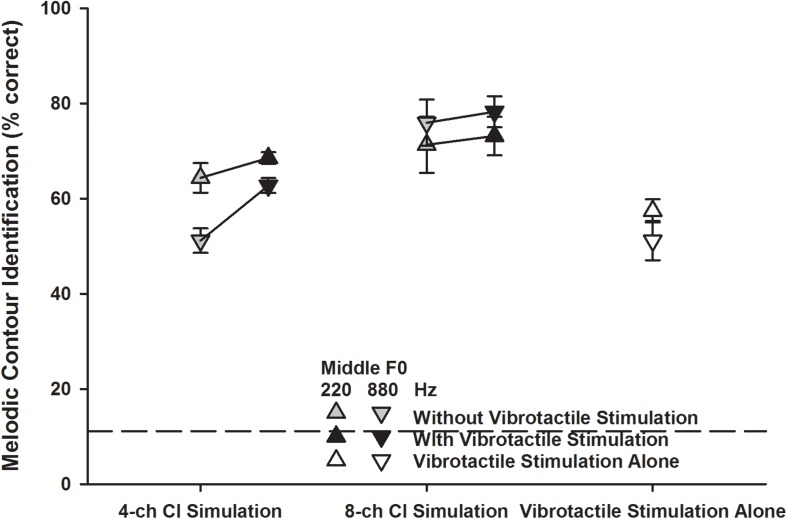
Melodic contour identification scores for the 220 and 880 Hz middle F0s (upward and downward triangles, respectively) with the 4- or 8-channel CI simulation (left and middle line plots, respectively) with or without vibrotactile stimulation (black and gray triangles, respectively) or with vibrotactile stimulation alone (white triangles). Symbols represent the mean, while error bars represent the standard error across participants. The horizontal dashed line indicates the chance performance of 11% correct.

Figure 5 shows the detailed MCI scores for the two middle F0s (220 Hz: upward triangles; 880 Hz: downward triangles) in the five experimental conditions. A two-way RM ANOVA was used to compare the performance for the 220 and 880 Hz middle F0s with vibrotactile stimulation alone (white triangles) to those with the 4- or 8-channel CI simulation alone (gray triangles). The middle F0 [*F*_(1, 7)_ = 12.91, *p* = 0.009] and experimental condition [*F*_(2, 14)_ = 12.58, *p* < 0.001] both significantly affected the MCI performance. There was a significant interaction between the two factors [*F*_(2, 14)_ = 11.15, *p* = 0.001]. *Post hoc* Bonferroni *t*-tests showed that for either the 220 or 880 Hz middle F0, the MCI performance with vibrotactile stimulation alone was similar to that with the 4-channel CI simulation alone (*p* > 0.42), but was significantly worse than that with the 8-channel CI simulation alone (*p* < 0.02). Also, the MCI performance with vibrotactile stimulation or 4-channel CI simulation alone was significantly better for the 220 Hz than for the 880 Hz middle F0 (*p* < 0.03), while the MCI performance with 8-channel CI simulation alone was similar for the two middle F0s (*p* = 0.09).

The detailed MCI scores for the 220 and 880 Hz middle F0s with the 4- or 8-channel CI simulation with or without vibrotactile stimulation (black and gray triangles, respectively) were analyzed using a three-way RM ANOVA with middle F0, channel number, and vibrotactile stimulation as the three factors. The significant effects of channel number and vibrotactile stimulation were the same as seen in the overall scores (Figure 4). The effect of middle F0 was not significant [*F*_(1, 7)_ = 3.76, *p* = 0.09] but there was a significant interaction between middle F0 and channel number [*F*_(1, 7)_ = 39.84, *p* < 0.001]. The other two- and three-way interactions were not significant (*p* > 0.09). To better understand how the MCI scores for the 220 and 880 Hz middle F0s changed with the channel number and vibrotactile stimulation, the MCI performance for each middle F0 was analyzed separately using a two-way RM ANOVA. When the middle F0 was 220 Hz, both the channel number [*F*_(1, 7)_ = 1.83, *p* = 0.22] and vibrotactile stimulation [*F*_(1, 7)_ = 1.63, *p* = 0.24] did not significantly affect the MCI performance, and the two factors did not have a significant interaction [*F*_(1, 7)_ = 0.20, *p* = 0.67]. When the middle F0 was 880 Hz, the MCI performance was significantly affected by both the channel number [*F*_(1, 7)_ = 36.11, *p* < 0.001] and vibrotactile stimulation [*F*_(1, 7)_ = 8.03, *p* = 0.02], and the two factors significantly interacted with each other [*F*_(1, 7)_ = 15.54, *p* = 0.006]. *Post hoc* Bonferroni *t*-tests showed that the MCI performance for the 880 Hz middle F0 was significantly better with 8 than with 4 channels (*p* < 0.002), whether or not vibrotactile stimulation was added to the CI simulations. On the other hand, simultaneous vibrotactile stimulation significantly improved the MCI performance for the 880 Hz middle F0 with 4-channel (*p* = 0.002) but not with 8-channel CI simulation (*p* = 0.41).

#### Detailed MCI Scores for Different Interval Sizes

**FIGURE 6:**
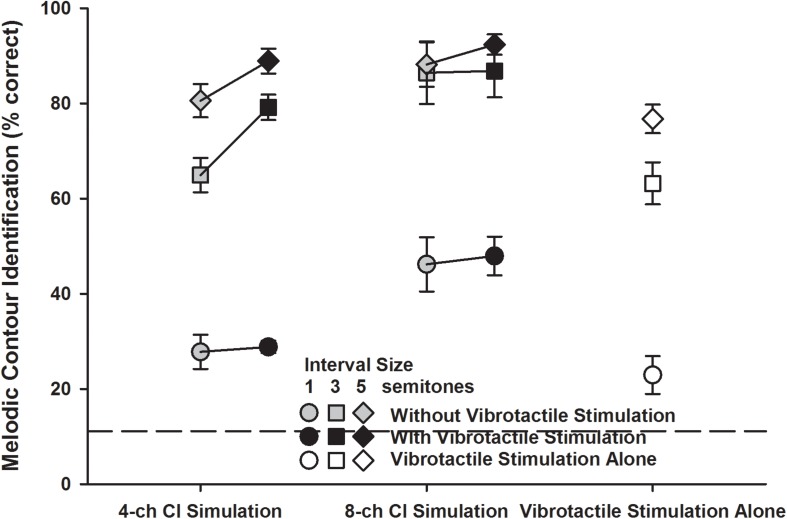
Melodic contour identification scores for the 1-, 3-, and 5-semitone intervals (circles, squares, and diamonds, respectively) with the 4- or 8-channel CI simulation (left and middle line plots, respectively) with or without vibrotactile stimulation (black and gray symbols, respectively) or with vibrotactile stimulation alone (white symbols). Symbols represent the mean, while error bars represent the standard error across participants. The horizontal dashed line indicates the chance performance of 11% correct.

Figure 6 shows the detailed MCI scores for the three interval sizes (1 semitone: circles; 3 semitones: squares; 5 semitones: diamonds) in the five experimental conditions. A two-way RM ANOVA was used to compare the performance for the 1-, 3-, and 5-semitone intervals with vibrotactile stimulation alone (white symbols) to those with the 4- or 8-channel CI simulation alone (gray symbols). Both the interval size [*F*_(2, 14)_ = 316.99, *p* < 0.001] and experimental condition [*F*_(2, 14)_ = 12.57, *p* < 0.001] significantly affected the MCI performance, but the two factors did not significantly interact with each other [*F*_(4, 28)_ = 1.88, *p* = 0.14]. *Post hoc* Bonferroni *t*-tests showed that for the 1- and 3-semitone intervals, the MCI performance with vibrotactile stimulation alone was similar to that with the 4-channel CI simulation alone (*p* = 1.00), but was significantly worse than that with the 8-channel CI simulation alone (*p* < 0.001). However, for the 5-semitone intervals, the MCI performance was similar with either the 8-channel CI simulation, 4-channel CI simulation, or vibrotactile stimulation alone (*p* > 0.12). With either the 4-channel CI simulation or vibrotactile stimulation alone, the MCI performance was significantly better for the 5-semitone than for the 3-semintone, and for the 3-semitone than for the 1-semitone intervals (*p* < 0.005). However, with the 8-channel CI simulation alone, the MCI performance was significantly better for the 3-semitone than for the 1-semitone intervals (*p* < 0.001), but was similar for the 3- and 5-semitone intervals (*p* = 1.00).

The detailed MCI scores for the 1-, 3-, and 5-semitone intervals with the 4- or 8-channel CI simulation with or without vibrotactile stimulation (black and gray symbols, respectively) were analyzed using a three-way RM ANOVA with interval size, channel number, and vibrotactile stimulation as the three factors. Again, the significant effects of channel number and vibrotactile stimulation were the same as seen in the overall scores (Figure 4). The effect of interval size was significant [*F*_(2, 14)_ = 183.41, *p* < 0.001] and there was a significant interaction between interval size and channel number [*F*_(2, 14)_ = 7.66, *p* = 0.006]. The other two- and three-way interactions were not significant (*p* > 0.09). Again, to better understand how the MCI scores for the 1-, 3-, and 5-semitone intervals changed with the channel number and vibrotactile stimulation, the MCI performance for each interval size was analyzed separately using a two-way RM ANOVA. For the 1-semitone intervals, the MCI performance was significantly affected by the channel number [*F*_(1, 7)_ = 15.19, *p* = 0.006] but not by vibrotactile stimulation [*F*_(1, 7)_ = 0.16, *p* = 0.69]. The two factors did not significantly interact with each other [*F*_(1, 7)_ = 0.01, *p* = 0.92]. For the 3-semitone intervals, the MCI performance was significantly affected by both the channel number [*F*_(1, 7)_ = 9.38, *p* = 0.02] and vibrotactile stimulation [*F*_(1, 7)_ = 17.64, *p* = 0.004], and the two factors also had a significant interaction [*F*_(1, 7)_ = 18.43, *p* = 0.004]. *Post hoc* Bonferroni *t*-tests showed that the MCI performance for the 3-semitone intervals was significantly better with 8 than with 4 channels with the CI simulations alone (*p* = 0.002) but not with the CI simulations plus vibrotactile stimulation (*p* = 0.17). Also, simultaneous vibrotactile stimulation significantly improved the MCI performance for the 3-semitone intervals with 4-channel (*p* < 0.001) but not with 8-channel CI simulation (*p* = 0.89). For the 5-semitone intervals, the effects of both channel number [*F*_(1, 7)_ = 4.77, *p* = 0.06] and vibrotactile stimulation [*F*_(1, 7)_ = 5.30, *p* = 0.05] on the MCI performance were barely significant. The two factors did not have a significant interaction [*F*_(1, 7)_ = 1.03, *p* = 0.34].

### Factors Affecting the MCI Improvement With Simulated Electro-Tactile Stimulation

**FIGURE 7:**
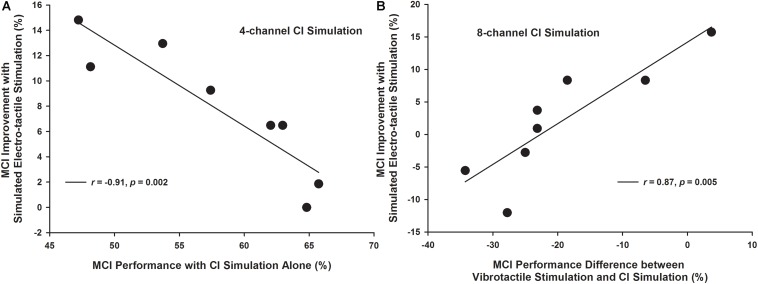
Melodic contour identification improvement with simulated electro-tactile stimulation as a function of the melodic contour identification performance with CI simulation alone (**A:** 4-channel CI simulation) and as a function of the melodic contour identification performance difference between vibrotactile stimulation and CI simulation (**B:** 8-channel CI simulation). Each line shows a linear regression with the corresponding correlation coefficient *r-* and *p*-value listed in the figure legend.

The improvement in MCI performance with simulated electro-tactile stimulation (i.e., the MCI performance with combined CI simulations and vibrotactile stimulation minus that with the CI simulations alone) varied across participants. To explain this variability, the vibration detection threshold, vibration discrimination performance averaged across different frequency intervals, MCI performance with vibrotactile stimulation alone, MCI performance with CI simulations alone, and MCI performance difference between vibrotactile stimulation and CI simulations were considered as potential factors contributing to the MCI improvement with simulated electro-tactile stimulation. Pearson correlation analyses with the Holm-Bonferroni correction showed that with the 4-channel CI simulation, the MCI improvement with simulated electro-tactile stimulation was significantly correlated with the MCI performance with CI simulation alone (*r* = −0.91, *p* = 0.002, Figure 7A). That is, participants having poorer MCI performance with the 4-channel CI simulation alone showed more MCI improvement with simulated electro-tactile stimulation. With the 8-channel CI simulation, the MCI improvement with simulated electro-tactile stimulation was significantly correlated with the MCI performance difference between vibrotactile stimulation and CI simulation (*r* = 0.87, *p* = 0.005, Figure 7B). That is, participants having less decline in MCI performance with vibrotactile stimulation alone than with the 8-channel CI simulation alone showed more MCI improvement with simulated electro-tactile stimulation. With the 8-channel CI simulation, the correlation between MCI improvement with simulated electro-tactile stimulation and MCI performance with the CI simulation alone just missed significance (*r* = −0.80, *p* = 0.018). The other factors were not significantly correlated with the MCI improvement with simulated electro-tactile stimulation (4-channel CI simulation: |*r|* < 0.42, *p* > 0.30; 8-channel CI simulation: |*r|* < 0.23, *p* > 0.58).

## Discussion

The present study used a compact, wearable vibrotactile device to produce F0-based vibrations at the forearm top of participants in real time and found significantly better MCI performance with than without vibrotactile stimulation of NH listeners listening to acoustic CI simulations. Specifically, the MCI improvement with simulated electro-tactile stimulation was significant with the 4-channel but not with the 8-channel CI simulation, for the 880 Hz but not for the 220 Hz middle F0, and for the 3- and 5-semitone but not for the 1-semitone intervals. The MCI improvement with simulated electro-tactile stimulation varied across participants, depending on the MCI performance with CI simulation alone or the MCI performance difference between vibrotactile stimulation and CI simulation.

To understand the different benefits of simulated electro-tactile stimulation to MCI performance with different channel numbers, middle F0s, and interval sizes, the effects of these factors on MCI performance with the CI simulation alone or vibrotactile stimulation alone will first be discussed. There was a significant interaction between middle F0 and channel number for the MCI performance with CI simulation alone. When the spectral resolution of CI simulation was limited to 4 channels, the MCI performance was significantly better for the 220 Hz than for the 880 Hz middle F0. It was because the temporal periodicity cues for pitch perception were available in the 500 Hz temporal envelopes of CI simulation for the 220 Hz but not for the 880 Hz middle F0. Increasing the spectral resolution of CI simulation to 8 channels significantly improved the frequency resolvability for the 880 Hz but not for the 220 Hz middle F0 (Galvin et al., 2007). The tradeoff between spectral and temporal cues (Luo et al., 2014a) may have led to similar MCI performance for the two middle F0s with the 8-channel CI simulation. On the other hand, the MCI performance with vibrotactile stimulation alone was significantly better for the 220 Hz than for the 880 Hz middle F0, which was unexpected because for both middle F0s, the melodic contours were transposed to the same low-frequency range of vibration. Although significant, the mean difference in MCI performance with vibrotactile stimulation alone between the two middle F0s was only 5%. More participants should be tested in the future to confirm this finding.

There was also some interaction between interval size and channel number for the MCI performance with CI simulation alone. With only 4 channels, the MCI performance significantly improved as the interval size between adjacent notes increased from 1 to 3 and then from 3 to 5 semitones. However, the MCI performance with 8 channels plateaued for the 3-semitone intervals, similar to the MCI results of real CI users (Galvin et al., 2007; Luo et al., 2018). Spectral analyses showed that the energy distribution across vocoder channels was more different for the 5-semitone than for the 3-semitone intervals with 4 channels but not with 8 channels. Increasing the spectral resolution of CI simulation from 4 to 8 channels significantly improved the MCI performance for the 1- and 3-semitone intervals and barely for the 5-semitone intervals. Pitch discrimination thresholds for the 220 Hz middle F0 with CI simulations have been shown to improve from 1.65 semitones with 4 channels to 1 semitone with 8 channels (Qin and Oxenham, 2005), which may explain the effect of channel number on MCI performance for the 1-semitone intervals. The present results were consistent with previous findings that reducing the channel interaction of a CI simulation significantly improved the MCI performance for 1- and 3-semitone intervals (Crew et al., 2012). Both studies showed the importance of spectral resolution to MCI. On the other hand, the MCI performance with vibrotactile stimulation alone was also significantly better for the 5-semitone than for the 3-semitone, and for the 3-semitone than for the 1-semitone intervals. This was likely due to the improved vibration discrimination with increasing frequency intervals as shown in the psychophysical studies of vibrotactile stimulation. Note that the MCI performance with vibrotactile stimulation alone was much worse than the vibration discrimination performance with the same frequency intervals. Each trial of MCI can be viewed as a series of four trials of adjacent note or vibration discrimination. It was thus not surprising that with the same frequency intervals, the MCI performance with vibrotactile stimulation alone (e.g., 23% correct for the 1-semitone intervals) was close to the vibration discrimination performance (e.g., 69% correct for the 1-semitone intervals) raised to the power of 4. Note that both the MCI and vibration discrimination tasks required the participants to judge the frequency change directions.

Overall, the MCI performance with vibrotactile stimulation alone was similar to that with the 4-channel CI simulation alone. The equal perceptual salience of both stimulation modes may have facilitated the multisensory integration for participants to have significantly better MCI performance with simulated electro-tactile stimulation than with the 4-channel CI simulation alone. In contrast, the MCI performance with vibrotactile stimulation alone was significantly worse than that with the 8-channel CI simulation alone. When vibrotactile stimulation was combined with the 8-channel CI simulation, the MCI performance may have been dominated by the more salient CI simulation signal, and thus did not significantly differ from that with the 8-channel CI simulation alone. The MCI improvement with simulated electro-tactile stimulation compared to the 4-channel CI simulation alone was significant for the 880 Hz but not for the 220 Hz middle F0. A possible explanation is that the vibration frequency cues used to represent the melodic contours may have shared a similar temporal mechanism with the envelope periodicity cues used for MCI around 220 Hz with the CI simulation, but were complementary to the spectral cues used for MCI around 880 Hz with the CI simulation. For example, the inter-spike interval code has been found in both the mechanoreceptive afferents for vibration frequency perception (e.g., Mountcastle et al., 1967) and in the auditory nerve fibers for pitch perception (e.g., Cariani and Delgutte, 1996). The MCI improvement with simulated electro-tactile stimulation compared to the 4-channel CI simulation alone was significant for the 3- and 5-semitone but not for the 1-semitone intervals, possibly because vibrotactile stimulation did not provide salient enough MCI cues for the 1-semitone intervals.

The inter-subject variability in MCI improvement with simulated electro-tactile stimulation was similar to that in speech recognition improvement (Huang et al., 2017; Fletcher et al., 2018). The correlation analyses showed that the ability to detect or discriminate vibrotactile stimuli or to identify the contour patterns of vibrotactile stimuli did not predict the amount of MCI improvement with simulated electro-tactile stimulation. Instead, the MCI performance with CI simulation alone, either by itself (when there were 4 channels) or relative to that with vibrotactile stimulation alone (when there were 8 channels), was significantly correlated with the MCI improvement with simulated electro-tactile stimulation. These results generally support the hypothesis that the relative salience of acoustic and vibrotactile stimulation cues determines the efficacy of multi-sensory integration and the simulated electro-tactile stimulation benefits to MCI. With only 4 channels, several participants had similar MCI performance with either the CI simulation or vibrotactile stimulation alone, and they had various amounts of MCI improvement with simulated electro-tactile stimulation, depending on the baseline performance with CI simulation alone (Figure 4). When there were 8 channels, participants had much more decline in MCI performance with vibrotactile stimulation alone than with the CI simulation alone, and such performance decline played an important role in determining the MCI improvement with simulated electro-tactile stimulation. Figure 7B shows that participants benefited from the combination of CI simulation and vibrotactile stimulation, as long as the MCI performance decline with vibrotactile stimulation alone compared to the CI simulation alone was less than 20%.

The present study extended previous research on speech recognition with electro-tactile stimulation (Huang et al., 2017; Fletcher et al., 2018) by showing that F0-based vibrotactile stimulation also improved music-related MCI performance with the 4-channel CI simulation. The current results were obtained with vibrotactile stimulation at the forearm top in order to represent real-world applications, although more MCI improvement may be expected if vibrotactile stimulation were applied to a more sensitive site such as the index fingertip as used in Huang et al. (2017) and Fletcher et al. (2018). Fletcher et al. (2018) found that training significantly improved the benefits of simulated electro-tactile stimulation to speech recognition in noise, but they only conducted training for the condition with combined CI simulation and vibrotactile stimulation. In contrast, all the conditions in the present study were tested after the same amount of brief training was provided to avoid a bias toward any condition. Future studies need to find the paradigm and duration of training needed for the most electro-tactile stimulation benefits.

The present study used a 4- or 8-channel noise-band vocoder to simulate the spectral resolution in real CI users (Friesen et al., 2001). The MCI performance of real CI users (Galvin et al., 2007; Luo et al., 2018) was similar to that with the 4-channel CI simulation alone but was worse than that with the 8-channel CI simulation alone. As such, real CI users may also receive benefits from F0-based vibrotactile stimulation for MCI, similar to the NH participants listening to the 4-channel CI simulation in the present study. The benefits of simulated electro-tactile stimulation to MCI were less than those of bimodal hearing in real CI users (Crew et al., 2015). The residual low-frequency acoustic hearing in bimodal CI users produced much better MCI performance than electric hearing with CIs (Crew et al., 2015), while vibrotactile stimulation produced similar MCI performance as the 4-channel CI simulation in the present study. Nevertheless, for CI users without residual low-frequency acoustic hearing, vibrotactile stimulation may be a viable option to improve pitch contour perception. While the number of participants in this study was small, we are currently testing a larger pool of CI users to determine whether the present CI simulation results may be generalized to real CI users. The benefits of electro-tactile stimulation to MCI may vary across CI users and training may be important to gauge and enhance such benefits for CI users.

For the vibration motor used in the present study, the vibration amplitude changed with the vibration frequency, meaning that the F0 extracted from each musical note in real time was encoded by both vibration amplitude and frequency. Co-varied acoustic amplitudes and frequencies have been shown to elicit common contour representations and thus significantly improve the MCI performance of CI users as compared to acoustic frequency changes alone (Luo et al., 2014a). It is also possible that contour identification may be enhanced with co-varied vibration amplitudes and frequencies. To separate the contributions of vibration amplitude and frequency cues to MCI performance, future studies may use a Linear Resonant Actuator (LRA) to independently control the vibration amplitude and frequency. F0-based vibrotactile stimulation may also improve other aspects of music perception such as melodic interval perception and familiar melody recognition, as well as voice pitch perception related to Mandarin tone, speech intonation, and vocal emotion recognition of CI users. These potential benefits of electro-tactile stimulation should be explored in future studies.

## Data Availability Statement

The datasets generated for this study are available on request to the corresponding author.

## Ethics Statement

This study was carried out in accordance with the recommendations of the Institutional Review Board of Arizona State University with written informed consent from all subjects. All subjects gave written informed consent in accordance with the Declaration of Helsinki. The protocol was approved by the Institutional Review Board of Arizona State University.

## Author Contributions

XL designed the experiments, supervised data collection, analyzed the data, and wrote the manuscript. LH participated in conceptualization of the project, designed the vibrotactile device, and contributed to manuscript writing.

## Conflict of Interest

The authors declare that the research was conducted in the absence of any commercial or financial relationships that could be construed as a potential conflict of interest.

## Footnotes

1 https://docs.sensestage.eu/sensestage-v1/overview-of-the-system

2 https://www.cycling74.com

3 https://www.precisionmicrodrives.com/product/datasheet/310-113-10mm-vibration-motor-3mm-type-datasheet.pdf
